# Supplementary material for: Ecosystem structural changes controlled by altered rainfall climatology in tropical savannas
Source: Nat Commun. 2019 Feb 8;10:671. doi: 10.1038/s41467-019-08602-6 (PMC6368604; doi:10.1038/s41467-019-08602-6)
Supplement: Supplementary file 1 — Supplementary Information [file 41467_2019_8602_MOESM1_ESM.pdf]

Supplementary information

Ecosystem structural changes controlled by altered rainfall climatology in tropical savannas

Zhang et al.

Study areas (global tropical savannas)

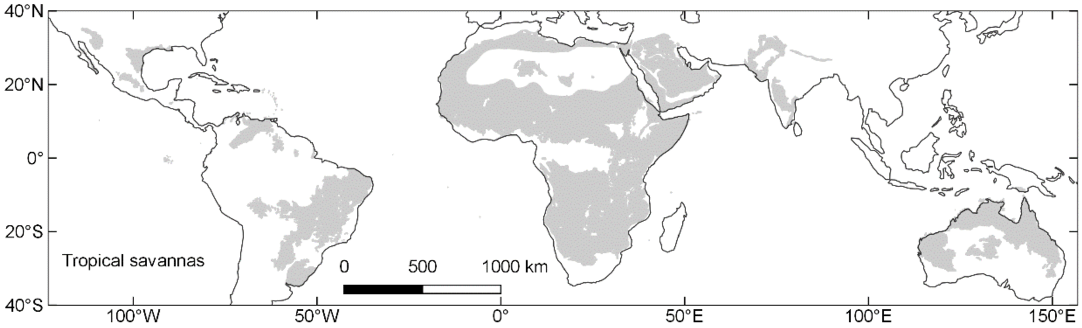

Supplementary Figure 1 | Map of tropical savannas (including xeric shrublands) defined by the World Wildlife Fund[1]. Global tropical areas refer to regions from 50°N to 50°S.

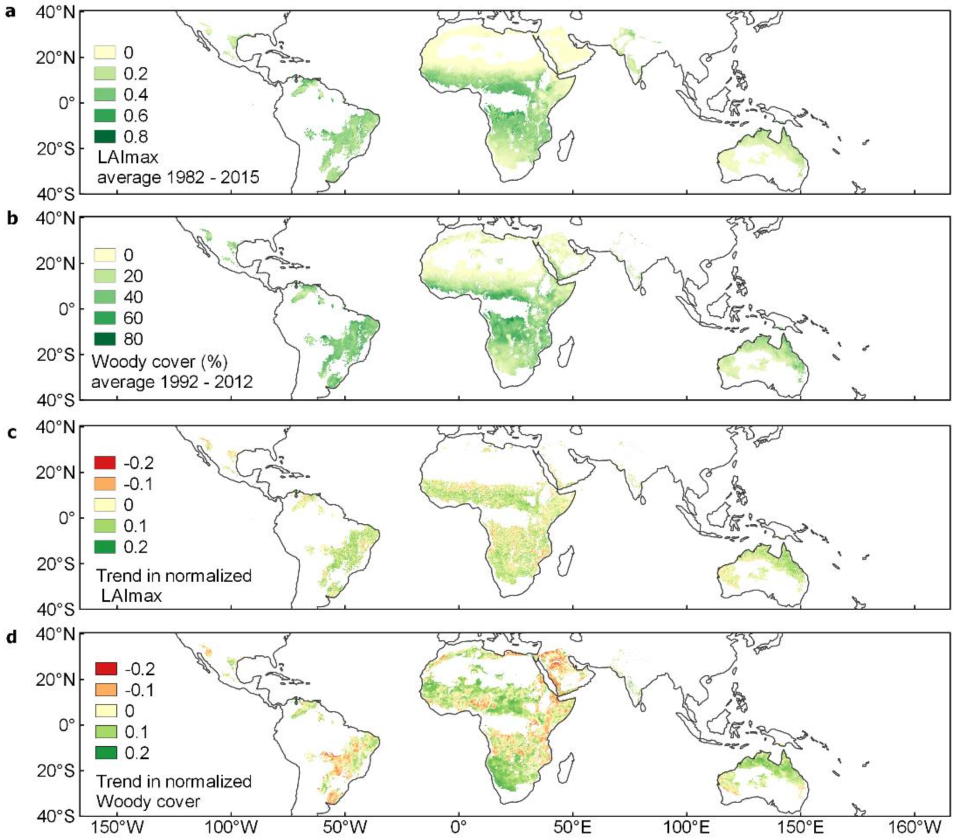

Supplementary Figure 2 | Spatial distribution of a, LAI_{max} averaged for 1982 – 2015, **b,** woody cover averaged for 1992 –2012, **c,** changes in normalized LAI_{max} and **d**, changes in normalized woody cover for 1992 – 2012,

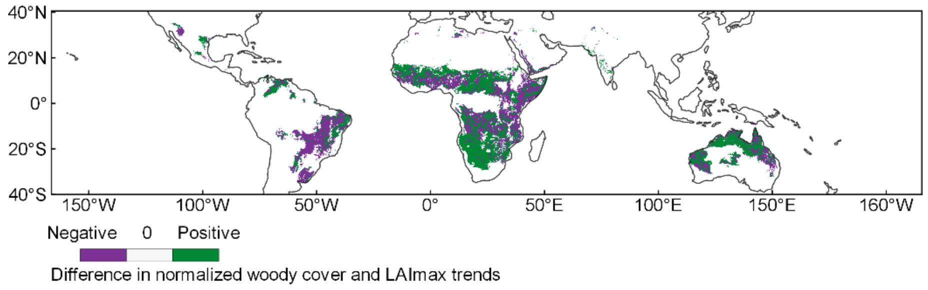

Supplementary Figure 3 | Differences in normalized woody cover and LAI_{max} trends. Green(/purple) colour represents areas where the normalized trend in woody cover is higher(/lower) than the trend of LAI_{max}.

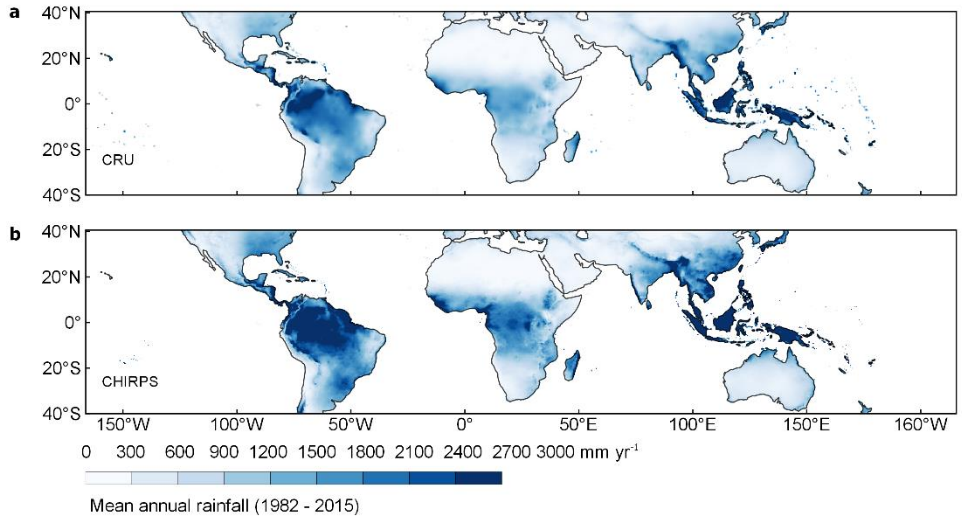

Supplementary Figure 4 | Mean annual rainfall based on a, CRU and **b,** CHIRPS datasets for 1982 – 2015.

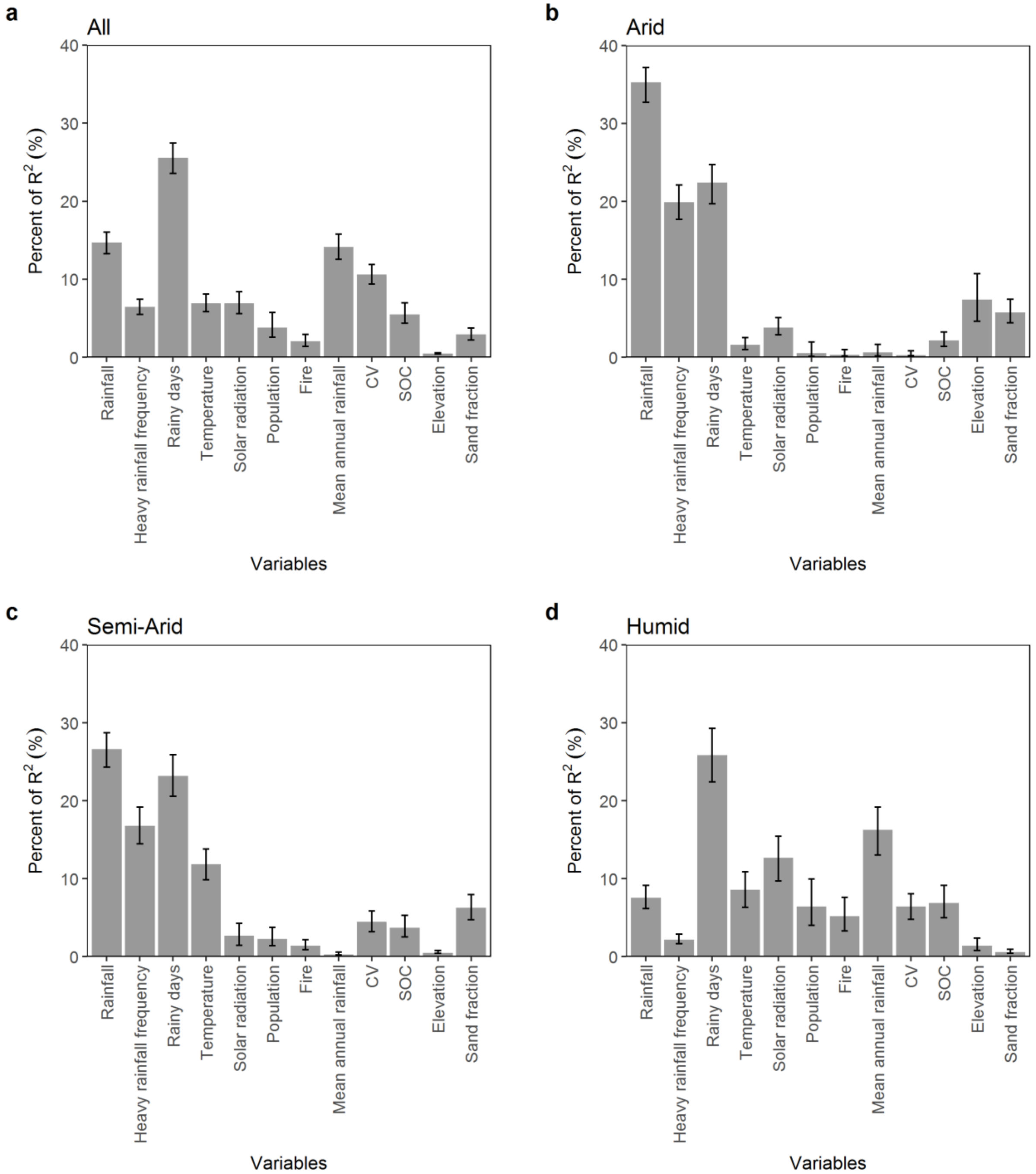

Supplementary Figure 5 | Relative weight of variables explaining changes in woody cover from the LMG (Lindeman, Merenda and Gold) method[2] based on a least squares regression model. The overall explaining power (R^2) of changes in woody cover, 1992-2012 (Based on VOD) for tropical savannas (All) and its sub-regions including arid (<300 mm yr^{-1}),

semi-arid (300 – 700 mm yr^{-1}) and humid regions (>700 mm yr^{-1}) by explanatory variables are 0.23, 0.63, 0.33 and 0.14, respectively. Explanatory variables are: change in annual rainfall (mm yr^{-1}), change in heavy rainfall frequency (events yr^{-1}), change in rainy days (days yr^{-1}), change in mean annual temperature (°C yr^{-1}) , change in solar radiation (expressed by cloud cover, % yr^{-1}); change in burned area fraction (Fire, % yr^{-1}); change in population density (persons km^{-2}), mean annual rainfall (mm yr^{-1}); coefficient of inter-annual rainfall variability (CV); soil organic carbon (SOC); elevation; sand fraction (%).

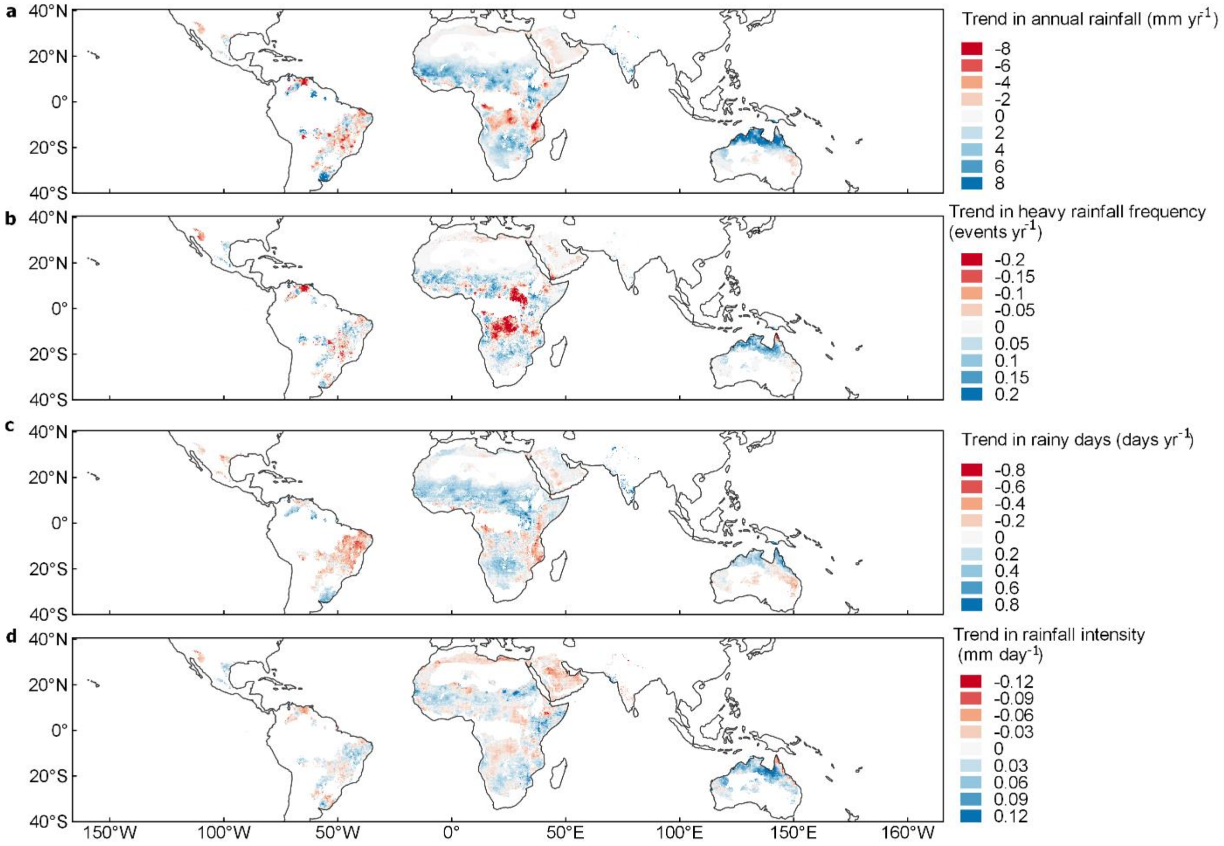

Supplementary Figure 6 | Spatial distribution of trends in rainfall climatology during 1982 – 2015. a, change in annual rainfall; **b,** change in heavy rainfall frequency; **c,** change in rainy days; **d,** change in rainfall intensity.

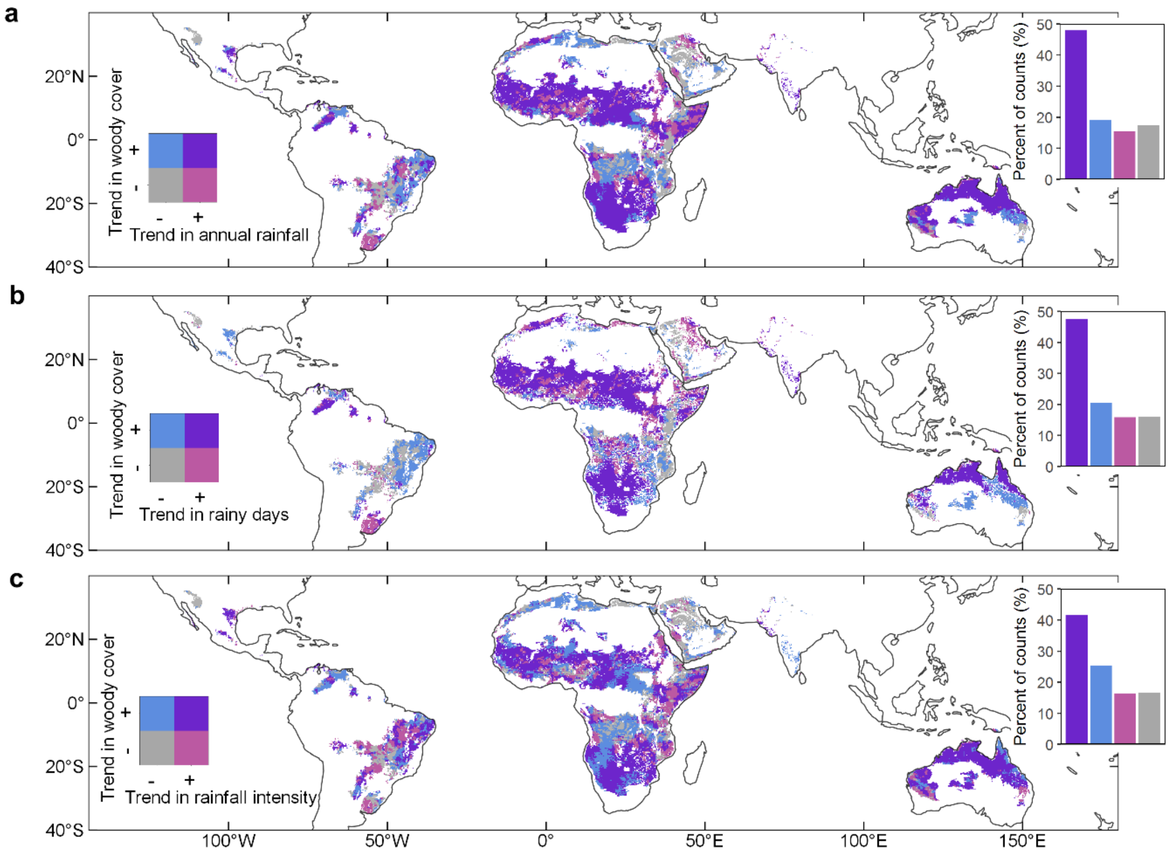

Supplementary Figure 7 | Spatial distribution of trends in rainfall climatology (1982 – 2015) and woody cover (1992 – 2012), a, trends in annual rainfall and woody cover; **b,** trends in rainy days and woody cover; **c,** trends in rainfall intensity and woody cover. Purple means increased trends in both rainfall climatology and woody cover, blue means an increased trend in woody cover but not in rainfall climatology, light red means an increased trend in rainfall climatology but not in woody cover, and grey means a decreasing trend in both rainfall climatology and woody cover. The bar plots show the percent of count for each group with the same colour scheme as the map (total number of pixels are 34 089, 28 757 and 34 154, respectively).

Supplementary Table 1 | Variance Inflation Factor (VIF) values for the explanatory variables to test for multi-collinearity. VIF < 5 means no multicollinearity among explanatory variables whereas VIF >5 indicate multicollinearity among explanatory variables. The study area is divided into tropical savannas (All) and its sub-regions including arid (<300 mm yr^{-1}), semi-arid (300 – 700 mm yr^{-1}) and humid regions (>700 mm yr^{-1}). Explanatory variables are: change in annual rainfall (mm yr^{-1}), change in heavy rainfall frequency (events yr^{-1}), change in rainy days (days yr^{-1}), change in rainfall intensity (mm day^{-1}), change in mean annual temperature (°C yr^{-1}) , change in solar radiation (expressed by cloud cover, % yr^{-1}); change in burned area fraction (% yr^{-1}); change in population density (persons km^{-2}), mean annual rainfall (mm yr^{-1}); coefficient of inter-annual rainfall variability (CV); soil organic carbon (SOC); elevation; Sand fraction (%). N indicates the number of pixels in each of the regions analysed.

VIF values	All	Arid (< 300 mm yr^{-1})	Semi-arid (300 – 700 mm yr^{-1})	Humid (> 700 mm yr^{-1})
	N = 17574	N= 1269	N = 4660	N = 11645
**Change in annual rainfall**	**9.03**	**14.51**	**11.25**	**8.49**
**Change in heavy rainfall frequency**	3.82	**5.59**	4.09	3.65
**Change in rainy days**	4.36	**7.47**	**6.11**	3.91
**Change in rainfall intensity**	**5.44**	**5.10**	**5.07**	**5.82**
**Change in temperature**	1.43	1.76	1.67	1.59
**Change in solar radiation**	1.13	1.50	1.16	1.21
**Change in population**	1.03	1.12	1.07	1.02
**Change in burned area fraction**	1.14	1.06	1.14	1.19
**Mean annual rainfall**	2.26	1.13	1.40	1.39
**CV**	2.54	1.22	1.74	1.83
**SOC**	1.15	1.63	1.49	1.09
**Elevation**	1.34	1.28	1.45	1.48
**Sand fraction**	1.26	1.79	1.70	1.23

Supplementary Table 2 | The overall explaining power (R^2) of a least squares regression model to explain changes in LAI_{max} for tropical savannas (All) and its sub-regions including arid (<300 mm yr^{-1}), semi-arid (300 – 700 mm yr^{-1}) and humid regions (>700 mm yr^{-1}), with explanatory variables identical to Supplementary Figure 5.

**Models**	**All**		**Arid (< 300 mm yr^{-1})**		**Semi-arid (300 – 700 mm yr^{-1})**		**Humid (> 700 mm yr^{-1})**	
	**N**	**R^2**	**N**	**R^2**	**N**	**R^2**	**N**	**R^2**
Least squares model	17574	0.011	1269	0.284	4660	0.108	11645	0.031

Supplementary Table 3 | Estimated coefficients of a least squares regression to predict changes in woody cover, 1992 – 2012 (Based on VOD) for tropical savannas (All) and its sub-regions including arid (<300 mm yr^{-1}), semi-arid (300 – 700 mm yr^{-1}) and humid regions (>700 mm yr^{-1}). Explanatory variables are identical to variables shown in Supplementary Table 1. N indicates the number of pixels in each run and the stars indicate statistical significance (**: p<0.05).

**Multiple regression**	**All**		**Arid**		**Semi-arid**		**Humid**	
	N = 17574		N = 1269		N = 4660		N = 11645	
	Estimate	Std.Error	Estimate	Std.Error	Estimate	Std.Error	Estimate	Std.Error
**Change in annual rainfall**	**0.037****	0.018	**0.554****	0.054	**0.133****	0.033	-0.019	0.023
**Change in heavy rainfall frequency**	**0.068****	0.015	0.055	0.041	**0.130****	0.025	0.024	0.019
**Change in rainy days**	**0.314****	0.013	**0.149****	0.037	**0.209****	0.021	**0.284****	0.017
**Change in temperature**	-0.088**	0.007	-0.097**	0.025	-0.110**	0.011	-0.108**	0.010
**Change in solar radiation**	0.135**	0.008	-0.049**	0.022	0.064**	0.013	0.156**	0.010
**Change in population**	-1.210**	0.091	-0.172	0.097	-0.442**	0.092	-1.144**	0.103
**Change in burned area fraction**	-0.142**	0.011	-0.268**	0.092	0.015	0.023	-0.149**	0.012
**Mean annual rainfall**	-0.294**	0.017	-0.054**	0.024	-0.004	0.009	-0.309**	0.020
**CV**	0.057**	0.014	-0.039	0.028	0.038**	0.018	0.038**	0.018
**SOC**	-0.573**	0.048	-0.011	0.042	-0.141**	0.047	-0.562**	0.054
**Elevation**	-0.023	0.015	0.153**	0.025	-0.028	0.016	-0.068**	0.021
**Sand fraction**	0.024**	0.008	0.027	0.024	0.075**	0.013	-0.027**	0.011

Supplementary Table 4 | Estimated coefficients of a partial regression model to predict changes in woody cover, 1992 – 2012 (Based on VOD) for tropical savannas (All) and its sub-regions including arid (<300 mm yr^{-1}), semi-arid (300 – 700 mm yr^{-1}) and humid regions (>700 mm yr^{-1}). Explanatory variables are identical to variables shown in Supplementary Table 1 and the stars indicate statistical significance (**: p<0.05).

Partial regression	All	Arid (< 300 mm yr^{-1})	Semi-arid (300 – 700 mm yr^{-1})	Humid (> 700 mm yr^{-1})
	Estimate	Estimate	Estimate	Estimate
**Change in annual rainfall**	**0.040****	**0.503****	**0.138****	-0.015
**Change in heavy rainfall frequency**	**0.067****	0.083**	**0.127****	0.021
**Change in rainy days**	**0.311****	**0.171****	**0.204****	**0.281****
**Change in temperature**	-0.088**	-0.098**	-0.109**	-0.104**
**Change in solar radiation**	0.135**	-0.044**	0.064**	0.155**
**Change in population**	-1.206**	-0.173	-0.439**	-1.136 **
**Change in burned area fraction**	-0.142**	-0.259**	0.016	-0.147**
**Mean annual rainfall**	-0.292**	-0.054**	-0.003	-0.307**
**CV**	0.058**	-0.035	0.040**	0.040**
**SOC**	-0.572**	-0.011	-0.143**	-0.559**
**Elevation**	-0.022	0.154**	-0.026	-0.067**
**Sand fraction**	0.024**	0.031	0.075**	-0.027**

Supplementary references:

1. Olson, D. M. *et al.* Terrestrial Ecoregions of the World : A New Map of Life on Earth. *BioScience* **51,** 933–938 (2001).
2. Grömping, U. Estimators of Relative Importance in Linear Regression Based on Variance Decomposition. *The American Statistician* **61,** 139–147 (2007).
